# The crustal geophysical signature of a world-class magmatic mineral system

**DOI:** 10.1038/s41598-018-29016-2

**Published:** 2018-07-13

**Authors:** Graham Heinson, Yohannes Didana, Paul Soeffky, Stephan Thiel, Tom Wise

**Affiliations:** 1 Department of Earth Sciences, University of Adelaide, Adelaide, SA 5005 Australia; 2 Geological Survey of South Australia, GPO BOX 320, Adelaide, SA 5001 Australia

## Abstract

World-class magmatic mineral systems are characterised by fluid/melt originating in the deep crust and mantle. However, processes that entrain and focus fluids from a deep-source region to a kilometre-scale deposit through the crust are unclear. A magnetotelluric (MT) and reflection seismic program across the margin of the Gawler Craton, Australia yield a distinct signature for a 1590 Ma event associated with emplacement of iron-oxide copper gold uranium (IOCG-U) deposits. Two- and three-dimensional MT modelling images a 50 km wide lower-crustal region of resistivity <10 Ωm along an accreted Proterozoic belt. The least resistive (~1 Ωm) part terminates at the brittle-ductile transition at ~15 km, directly beneath a rifted sedimentary basin. Above the brittle-ductile transition, three narrow low-resistivity zones (~100 Ωm) branch to the surface. The least resistive zone is remarkably aligned with the world-class IOCG-U Olympic Dam deposit and the other two with significant known IOCG-U mineral occurrences. These zones are spatially correlated with narrow regions of low seismic reflectivity in the upper crust, and the deeper lower-crust conductor is almost seismically transparent. We argue this whole-of-crust imaging encapsulates deep mineral system and maps pathways of metalliferous fluids from crust and mantle sources to emplacement at discrete locations.

## Introduction

**Figure 1:**
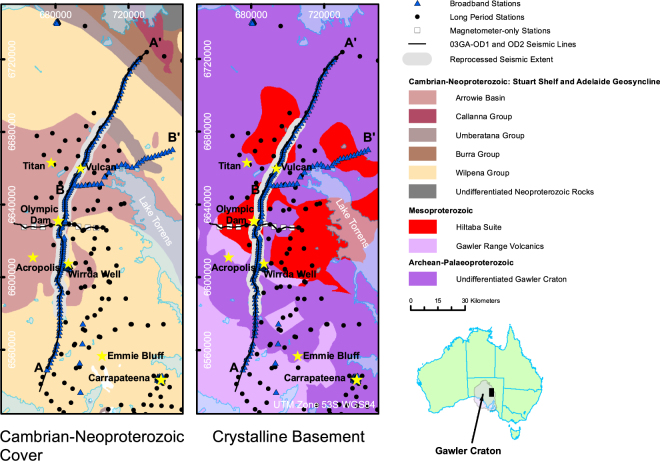
Location map of the survey area showing Neoproterozoic-Cambrian sedimentary cover (left hand side); crystalline basement geology (right hand side). Yellow stars are major mines and mineral occurrences: most notable along the transect are Acropolis-Wirrda Well; Olympic Dam; and Vulcan-Titan. Carapateena is a major IOCG-U mineral deposit under development as a new mine. Blue triangles show broadband MT sites; black circles show long period MT sites; and white squares show magnetometer-only sites. The black lines (03GA-OD1 and OD2) are the original seismic reflection transects^16^, and the reprocessed section is shown by the wider grey band^17^. Figure 1 maps were created using ArcGIS 10.3.1 software (https://www.esri.com/arcgis/about-arcgis).

World-class magmatic ore systems are often characterised by fluids/melts that are derived from deep lithosphere, mostly located at the margins of ancient craton^1–6^. There is, however, debate about the source of metals, and how they migrate from deep crust and upper mantle to focus as kilometre-scale deposits in the upper crust^3^. The Olympic Dam mine in South Australia lies beneath 300 m of cover and is one of the largest IOCG-U deposits globally: it is currently the fourth largest copper and the largest known deposit of uranium in the world^7^. The mine is in Paleoproterozoic crust under Neoproterozoic sediments of the Stuart Shelf, marginal to the Archean core of the Gawler Craton (Fig. 1). Most of the magmatic ore metals have been dated at 1590 Ma, and associated with the widespread Mesoproterozoic Hiltaba volcanism and silicic-dominated Gawler Range Volcanic large igneous province^8–12^, but there is evidence of post Hiltaba U mineralization dating to 1100 Ma^13^. Since these events, the lithosphere appears to have experienced little subsequent deformation so that the crust has remained largely undisturbed for over a billion years^14,15^. From potential field geophysics, drilling and current mining it is evident that hydrothermal mineralisation extends well below 2 km depth, but the full vertical extent of the system, and its connection to deeper sources is poorly known^7,8^.

In 2003, a deep seismic reflection traverse^16,17^ was conducted over a 200 km transect centred on the Olympic Dam mine to elucidate the deeper structural geological setting, as shown in Fig. 1. At the same time, a long-period (10^1^–10^4^ s bandwidth) MT transect was collected along the seismic transect with site spacing of 5 km^18^. Two-dimensional inversion of the MT responses revealed a significant conductive structure (1–10 Ωm) at mid to lower crustal depths, with a pronounced conductive region aligned with the occurrence of Olympic Dam at the surface^18^. Additional long-period MT sites were acquired in an approximate grid formation with 10 km spacing in 2009–2010, and a total of 110 broadband MT sites (periods of 10^−3^–10^3^ s) along the seismic transect and an adjacent transect with site spacing of 1–2 km in 2015–2016.

## Results

**Figure 2:**
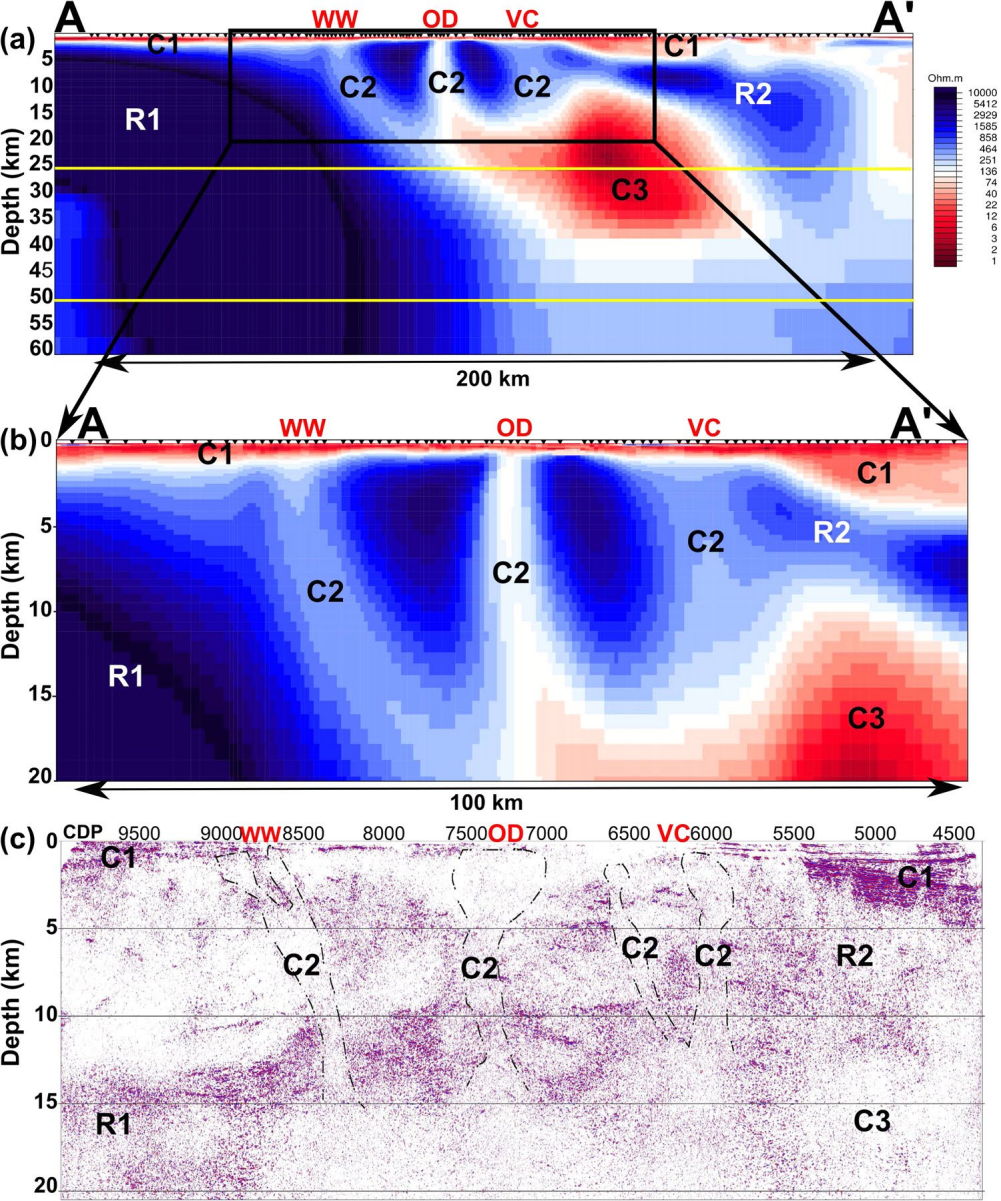
(**a**) 2D resistivity model of Profile A-A’ to a depth of 60 km. (**b**) The central part of the profile is expanded to a depth of 20 km. The Archean Gawler Craton on the left-hand side, and Proterozoic mobile belt on the right-hand side are characterized by very high resistivity (blue colour, R1 and R2) to a depth of more than 60 km. A striking high conductivity structure (C3) is situated at the margins of the Archean Gawler Craton at a depth 15–40 km in the mid to lower crust. In addition, three narrow low-resistivity pathways (C2) extend from conductor C3 to the surface, which link the lower crust with major IOCG-U mineral deposits. (**c**) 2D Seismic depth converted image^17^ showing zones of reduced reflectivity (C2 and C3) under all major mineral deposits. WW, OD and VC denote the major occurrences at Wirrda Well, Olympic Dam and Vulcan, respectively as shown in Fig. 1.

Two-dimensional resistivity inversion^19^ of MT data reveals a conductive surface layer (C1) associated with Neoproterozoic basin sediments overlain by transported Tertiary cover, that is similarly resolved in the reflection seismic section, as shown in Fig. 2. The cover thickness is least around the Olympic Dam deposit at about 300 m (consistent with drilling)^7^, but thickens significantly in a deep-rifted basin to the north-east, with sediment thickness >2 km. Minor resistivity variations within the sediments along the profile can be attributed to porosity differences and clay content.

The Archean Gawler Craton in the south west of the profile is characterized by high resistivity (>1000 Ωm, R1) and high seismic reflectivity to a depth of more than 60 km. The Proterozoic crust at the northern eastern end exhibits similar characteristics with high resistivity (R2). Between these regions, a low-resistivity <10 Ωm 50-km wide structure (C3) is imaged along the margin of the Archean Gawler Craton at a depth of 15–40 km in the mid to lower crust. This C3 conductor is coincident with a region of low seismic reflectance^16,18^, and lies directly beneath the deep-rifted Neoproterozoic basin C1 to the north east of Olympic Dam, suggesting that this portion of the crust has rheological weakness. The existence of a highly conducting lower crust at C3 was previously noted, and was clearly evident in both the long-period MT responses and the vertical field anomalies^18^.

Three narrow low-resistivity ~100 Ωm (C2) pathways extend from the top of the conductive region at the brittle-ductile transition depth of ~15 km to the base of the sedimentary layer C1 and align remarkably with the spatial location of the major IOCG-U mineral deposits at Wirrda Well (WW), Olympic Dam (OD) and Vulcan (VC) that are adjacent to the transect (Figs 1 and 2). These features are also apparent in the reprocessed seismic section as breaks in reflectance horizons with overall low reflectivity^17^ (Fig. 2). We note that these C2 features are not spatially aligned with significant mapped crustal faults^16^.

**Figure 3:**
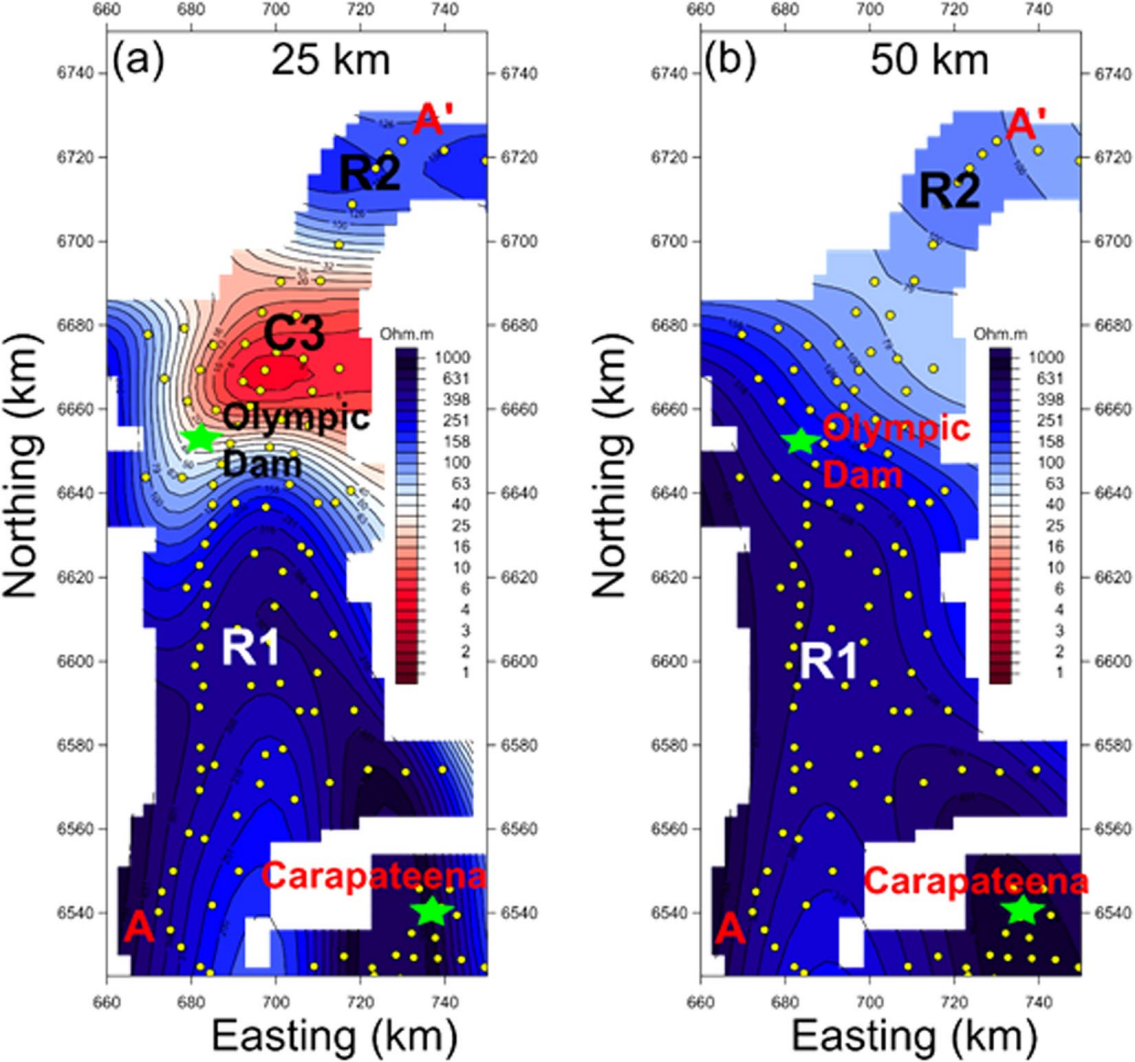
3D resistivity maps at depths of (**a**) 25 km (**b**) 50 km reveal that most of the Archean Gawler Craton in the south west is characterized by high resistivity structure in the lower crust. At 25 km depth, low resistivity is imaged to the north-east of the Olympic Dam deposit. C3 is a low resistivity zone and R1, R2 are high resistivity zones associated with the Archean Gawler Craton and Proterozoic mobile belt, as shown in Fig. 2.

To provide regional context to the 2D model sections, we inverted gridded long-period MT responses (black circles in Fig. 1) using the 3D inversion code of Mackie, *et al*.^20^. The 3D inversion consisted of the full impedance tensor and vertical magnetic transfer function data from 152 stations for 19 periods in the bandwidth 10^1^–10^4^ s. The model space extends 2400 km × 2200 km × 1000 km in N-S, E-W and vertical directions, respectively. Resolution is coarse in the top 10 km due to the inherent sensitivity of long-period MT data and average site spacing of 10 km or more, and thus the fine-scale conductive features C2 are not resolved. However, mid to lower crustal resistivity changes over scale lengths greater than 10 km are better constrained, and the preferred 3D resistivity model depth slices at 25 and 50 km are shown in Fig. 3. The 25 km section images a low-resistivity C3 structure to the north east of Olympic Dam, consistent with the 2D model section in both depth, location and strike (Fig. 3(a): fits of MT and tipper data are shown in Supplementary Fig. S6). At 50 km in Fig. 3(b) the low-resistivity region is not imaged in the uppermost mantle, although with all MT models it is difficult to infer much below a conductive lower crust.

## Discussion

Melts and fluids released from a mantle thermal perturbation in a form of a plume^21^ or delamination of the sub continental lithospheric mantle propagate upwards utilising a Moho offset as imaged in the seismic transect^16,17^. Metalliferous fluids then reach a rheological barrier ponding beneath the brittle-ductile boundary, before migrating to the surface by hydro-fracturing where hydrostatic pressures are greater than lithostatic pressure^22^. The high seismic reflectivity zone above and in-between the conductive pathways reflects the brittle-ductile transition itself, which is known to have a quick succession of strong and weak layers separated by mid-crustal detachment faults^23^. This zone (R1) also dips towards the colder Craton where heat flow is significantly lower and the temperature cooler.

Geochemical evidence for a larger mafic or mantle input is supported from studies of Rare Earth Elements (REE) and metals at Olympic Dam that show a mafic or mantle input (or abundance of crustal reworking, based on the Nd isotope data)^8^. There is evidence for an oxidising fluid based on sulphur isotope studies^10^ with a source either from hydrothermal cells in sediments^11,24^ or crustal rocks^9,10^. There is a good argument to be made that there are two sources of melts/fluids, especially during the 1590 Ma event^14^: one is mantle-derived and the other is crustal. The mantle connection is also manifested through the presence of olivine-phyric basalts intersected at Olympic Dam and Wirrda Well, suggesting a heterogeneous mantle source^12^.

The crust to the north of Olympic Dam has high heat flow >100 mW/m^2^. A possible causal mechanism for the conductor C3 may thus be associated with elevated lower-crustal temperatures >700 °C due to high-concentrations of radiogenic heat sources in the upper crust^25–27^, coupled with the presence of hydrated minerals as a results of fluid and magma migration from the lower most crust and upper mantle to the brittle-ductile zone^28^. Similar electrically conductive lithospheric structures were revealed in layered intrusive complexes of the Slave^29^ and Kaapvaal Cratons^30^ and for a world-class gold deposit in the Barberton Greenstone Belt^31^.

The path of the crustal fluids from lower-crustal source is mapped out by the 2D resistivity model as the conductive “fingers” C2 and in the seismic data as zones of reduced reflectivity that are spatially correlated with known IOCG-U mineral occurrences. Although there may be some free meteoric-derived fluids in brecciated haematite it is unlikely that significant porosity persists to mid-crustal depths. Ore-bearing magma and fluids began in a highly oxidising state when sourced from the mantle^32^. It is probable that upper crustal resistivities of order 100 Ωm result from conducting phases at grain boundaries most likely sulphides due to the oxidising signature from sulphur isotope analysis^10^, with metal precipitation in the uppermost crust due to a mixing of a meteoric fluid with a deep oxidising fluid.

## Methods

Dimensionality analyses of the MT data were carried out using the phase tensor approach^33^ and the ellipticity criterion^34^. Most of the responses were 1D at short periods (<1 s), and 2D at periods of 10^0^–102 s and generally up to 10^3^ s. Strike analyses using the azimuth of phase tensor^33^ and invariants of impedance tensor^35^ revealed consistent strike of N115°E for periods 10^0^–10^2^ s (Supplementary Fig. S1). This orientation is consistent with the strike of the long-wavelength gravity (shown in Supplementary Fig. S2). Phase tensor skew angles are less than five degrees for almost all sites at periods <10^3^ s, consistent with a predominantly 2D regional resistivity structure (Supplementary Figs S2 and S3).

On the basis of the dimensionality analysis, we inverted all rotated MT responses (to a strike of N115°E) in the bandwidth of 10^−3^ to 10^2^ and selectively 10^3^ s along profile A-A’ projected onto a transect with orientation N25°E using the 2D algorithm of Rodi and Mackie^19^ implemented in the WinGlink package (Profile B-B’ in Fig. 1 is shown in the Supplementary Fig. S7). The model presented was generated from a 100 Ωm half-space start, optimal smoothing parameter of $\tau$ = 1, and error floors of 5% and equivalently 1.43 degrees for resistivity and phase angle, respectively (Fig. 2, fits of model and data for selective sites are given in Supplementary Fig. S4). The final misfit for the model was RMS = 1.8, with largest misfits for the TE mode at periods greater than 10^2^ s. Static shifts on each mode of apparent resistivity were inverted for as an independent variable at each site but were found to be small (less than half an order of magnitude) for all 110 sites due to the uniform conductive cover (Supplementary Fig. S5).

We inverted an array of long-period MT stations with 5–10 km site spacing, in a 50 km swath around transects A-A’ and B-B’ using a 3D inversion code of Mackie *et al*.^20^ as a check on the plausibility and limitations of the 2D model. The 3D inversion included the full impedance tensor and vertical magnetic transfer function data from 152 stations at 19 periods in the bandwidth of 10^1^ to 10^4^ s. The model space extends 2400 km by 2200 km by 1000 km in NS, EW and vertical directions, respectively, and the grid was discretised into 290 × 110 × 58 cells. Thickness of the first layer is 250 m and increased by a factor of 1.1 for subsequent layers. The starting model was a 100 Ωm half-space. Static shifts were not included in the modelling but are generally small and can be accommodated by thin near-surface mesh blocks between sites. Error floors of 3%, 30% and 0.03 were used for the off-diagonal impedances, diagonal impedances and tipper, respectively. The RMS fit to all data was 1.7, selected sites of observations and model responses for both apparent resistivity and phase, and for tipper are shown in Supplementary Fig. S6.

### Data availability

Original MT response functions in Electrical Data Interchange (EDI) format and seismic reflection data from line 03GA-OD1 in the SEGY format and as images are freely available from the South Australian Resources Information Gateway (SARIG) platform from the Government of South Australia (https://map.sarig.sa.gov.au/).

## Electronic supplementary material

Supplementary Information

## References

[1] Begg GC (2010). Lithospheric, cratonic, and geodynamic setting of Ni-Cu-PGE sulfide deposits. Economic Geology.

[2] Richards JP (2011). Magmatic to hydrothermal metal fluxes in convergent and collided margins. Ore Geology Reviews.

[3] Richards JP (2013). Giant ore deposits formed by optimal alignments and combinations of geological processes. Nature Geoscience.

[4] Richards JP, Mumin AH (2013). Magmatic-hydrothermal processes within an evolving Earth: Iron oxide-copper-gold and porphyry Cu± Mo± Au deposits. Geology.

[5] Griffin WL, Begg GC, O’Reilly SY (2013). Continental-root control on the genesis of magmatic ore deposits. Nature Geoscience.

[6] Tassara S (2017). Plume-subduction interaction forms large auriferous provinces. Nature Communications.

[7] Ehrig, K., McPhie, J. & Kamenetsky, V. S. In Geology and Genesis of Major Copper Deposits and Districts of the World: A Tribute to Richard H. Sillitoe Vol. 16 (eds Hedenquist J. W., M. Harri, & F. Camus) 237–267 (Society of Economic Geologists, Inc., 2012).

[8] Johnson J, Cross K (1995). U-Pb geochronological constraints on the genesis of the Olympic Dam Cu-U-Au-Ag deposit, South Australia. Economic Geology.

[9] Skirrow RG (2007). Timing of iron oxide Cu-Au-(U) hydrothermal activity and Nd isotope constraints on metal sources in the Gawler Craton, South Australia. Economic Geology.

[10] Bastrakov EN, Skirrow RG, Davidson GJ (2007). Fluid evolution and origins of iron oxide Cu-Au prospects in the Olympic Dam district, Gawler Craton, South Australia. Economic Geology.

[11] McPhie J, Kamenetsky VS, Chambefort I, Ehrig K, Green N (2011). Origin of the supergiant Olympic Dam Cu-U-Au-Ag deposit, South Australia: Was a sedimentary basin involved?. Geology.

[12] Huang Q (2016). Olivine-phyric basalt in the Mesoproterozoic Gawler silicic large igneous province, South Australia: Examples at the Olympic Dam Iron Oxide Cu–U–Au–Ag deposit and other localities. Precambrian Research.

[13] Kirchenbaur M (2016). Uranium and Sm isotope studies of the supergiant Olympic Dam Cu–Au–U–Ag deposit, South Australia. Geochimica et Cosmochimica Acta.

[14] Reid AJ, Payne JL (2017). Magmatic zircon Lu–Hf isotopic record of juvenile addition and crustal reworking in the Gawler Craton, Australia. Lithos.

[15] Hand M, Reid A, Jagodzinski L (2007). Tectonic framework and evolution of the Gawler Craton, Southern Australia. Economic Geology.

[16] Drummond B, Lyons P, Goleby B, Jones L (2006). Constraining models of the tectonic setting of the giant Olympic Dam iron oxide–copper–gold deposit, South Australia, using deep seismic reflection data. Tectonophysics.

[17] Wise T (2015). Olympic Dam seismic revisited: reprocessing of deep crustal seismic using partially preserved amplitude processing. MESA Journal.

[18] Heinson GS, Direen NG, Gill RM (2006). Magnetotelluric evidence for a deep-crustal mineralizing system beneath the Olympic Dam iron oxide copper-gold deposit, southern Australia. Geology.

[19] Rodi W, Mackie RL (2001). Nonlinear conjugate gradients algorithm for 2-D magnetotelluric inversion. Geophysics.

[20] Mackie, R. L., Rodi, W. & Watts, M. D. In SEG Technical Program Expanded Abstracts 2001 1501–1504 (Society of Exploration Geophysicists, 2001).

[21] Thiel, S. & Heinson, G. Electrical conductors in Archean mantle—Result of plume interaction? *Geophysical Research Letters*, 10.1002/grl.50486 (2013).

[22] Connolly, J. A. D. & Podladchikov, Y. Y. Fluid flow in compressive tectonic settings: Implications for midcrustal seismic reflectors and downward fluid migration. *Journal of Geophysical Research: Solid Earth* **109**, 10.1029/2003JB002822 (2004).

[23] Regenauer-Lieb K, Weinberg RF, Rosenbaum G (2006). The effect of energy feedbacks on continental strength. Nature.

[24] Cherry AR (2017). Linking Olympic Dam and the Cariewerloo Basin: Was a sedimentary basin involved in formation of the world’s largest uranium deposit?. Precambrian Research.

[25] Beardsmore, G. R. & Cull, J. P. *Crustal heat flow: a guide to measurement and modelling*. (Cambridge University Press, 2001).

[26] Sandiford M, McLaren S (2002). Tectonic feedback and the ordering of heat producing elements within the continental lithosphere. Earth and Planetary Science Letters.

[27] McLaren S, Sandiford M, Powell R (2005). Contrasting styles of Proterozoic crustal evolution: A hot-plate tectonic model for Australian terranes. Geology.

[28] Yang X (2011). Origin of high electrical conductivity in the lower continental crust: a review. Surveys in Geophysics.

[29] Jones AG (2003). The electrical structure of the Slave Craton. Lithos.

[30] Evans RL (2011). Electrical lithosphere beneath the Kaapvaal Craton, southern Africa. Journal of Geophysical Research: Solid Earth.

[31] de Wit M (2017). Paleoarchean bedrock lithologies across the Makhonjwa Mountains of South Africa and Swaziland linked to geochemical, magnetic and tectonic data reveal early plate tectonic genes flanking subduction margins. Geoscience Frontiers.

[32] Wood BJ, Bryndzia LT, Johnson KE (1990). Mantle oxidation state and its relationship to tectonic environment and fluid speciation. Science.

[33] Caldwell TG, Bibby HM, Brown C (2004). The magnetotelluric phase tensor. Geophysical Journal International.

[34] Becken M, Burkhardt H (2004). An ellipticity criterion in magnetotelluric tensor analysis. Geophysical Journal International.

[35] Weaver J, Agarwal A, Lilley F (2000). Characterization of the magnetotelluric tensor in terms of its invariants. Geophysical Journal International.

