# Supplementary material for: The crustal geophysical signature of a world-class magmatic mineral system
Source: Sci Rep. 2018 Jul 13;8:10608. doi: 10.1038/s41598-018-29016-2 (PMC6045595; doi:10.1038/s41598-018-29016-2)
Supplement: Supplementary file 1 — Supplementary Information [file 41598_2018_29016_MOESM1_ESM.pdf]

The crustal geophysical signature of a world-class magmatic mineral system

Supplementary information

Graham Heinson[1], Yohannes Didana[1], Paul Soeffky[1], Stephan Thiel[1,2], Tom Wise[2]

[1]Department of Earth Sciences, University of Adelaide, Adelaide, SA 5005, Australia

[2]Geological Survey of South Australia, Adelaide, SA 5000, Australia

Seismic data processing

A variation of standard seismic processing methodology known as partially preserved amplitude processing was applied to the original reflection data from seismic line 03GA-OD1[1,2]. In contrast with the original processing[2] of line 03GA-OD1 where workflow was designed to provide a consistent image of the whole crust, this processing technique emphasises variation in signal strength by preserving the temporal relative amplitude ratio, which has the effect of sharpening the output image, particularly in the upper crust.

Wise et al.[1] compared the results of the differing processing methodologies, and concluded that greater reflectivity contrasts in the upper crust are revealed by this approach, permitting interpretation of steeply dipping seismic structures and weakly-reflective zones which may reflect possible fossil fluid pathways.

MT data processing

Magnetotellurics is natural-source EM method used to explore the resistivity distribution of Earth on scales of tens of metres to hundreds of kilometres[3]. In the MT method, orthogonal components of inducing horizontal magnetic fields (**H**) and resulting horizontal electric fields

(**E**) are measured simultaneously as a function of time to determine the Earth's electrical impedance as a function of frequency. The fields are related, in the frequency domain, by the impedance tensor (**Z**) given by **E**=**ZH**. Apparent resistivity ρ_a as a function of frequency *f* is given by $\rho_a = \frac{1}{\mu_0 \omega}|Z|^2$, where Z is an impedance element, μ_o is magnetic permeability of free space, ω is angular frequency $\omega = 2\pi f$. The skin-depth δ (approximate depth of investigation in kilometres) is $\delta(T) \approx 0.5\sqrt{T\rho_a}$ where ρ_a is the apparent resistivity, or the average resistivity of an equivalent half-space, and T is the period of induction. The complex impedance tensor can be written in terms of its real (**X**) and imaginary (**Y**) parts as **Z** = **X** + i **Y**, from which the MT phase tensor is defined by the relation $\mathbf{\Phi} = \mathbf{X}^{-\mathbf{1}}\boldsymbol{Y}$ and is not affected by galvanic distortion [4,5].

The Olympic Dam transect A-A' in Figure 1 consisted of a 200 km transect of 110 broadband sites with a 1 to 2 km spacing and 30 long-period instruments at 5 km intervals[6]. An additional thirty broadband sites along B-B' were deployed to form a northeast-southwest transect just north of Olympic Dam at 2 km intervals. The MT data were processed using a robust, remote-reference code[7], resulting in response functions over the period range of 10^1 - 10^4 s for long-period sites and 10^{-3} – 10^3 s for broadband sites.

Strike analyses using the azimuth of phase tensor[4] and invariants of impedance tensor[8] approaches revealed a dominant geo-electric strike of N115°E at periods >1 s, as shown in Figure S1. At short periods (<1 s), there is no well-defined strike as induction is predominantly in the sedimentary cover which is 1D. We note that the longer-period geoelectric strike is consistent with the strike of the long-wavelength Bouguer gravity trends in Figure S2, and although there is a 90 degree ambiguity from the tensor alone, the geology indicates that this is the correct modelling orientation.

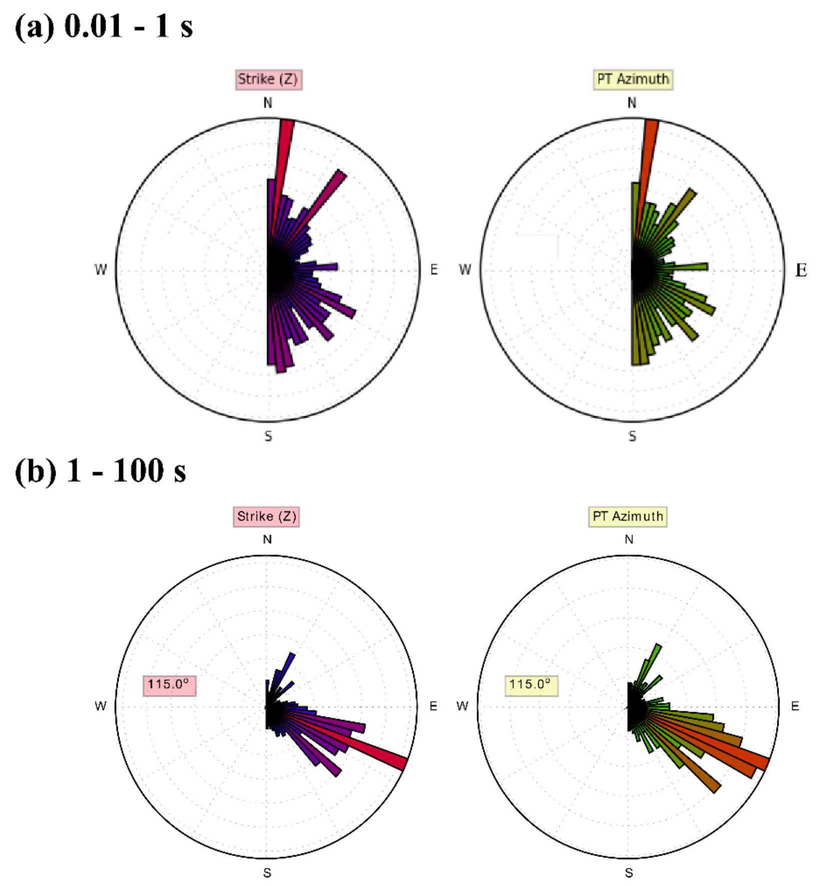

Figure S1: Rose diagram of strike angles (a) at periods < 1 s with no defined direction, consistent with a predominantly 1D sedimentary structure; (b) at periods of 1-100 s showing dominant strike of N115°E estimated from the invariants of the impedance tensor (left hand figures)[8] and the azimuth of phase tensor (right hand figures)[4]. Warmer colours (reds) indicate higher numbers of occurrence; greens and purples show lower occurrences.

MT modelling

All MT responses were rotated to this geoelectric orientation and the modelled transect was N25°E (at right angles to the geoelectric strike). In this rotated frame, the orientation of the impedance component with magnetic field parallel to geoelectric strike is denoted the TM mode, and the impedance component with the electric field parallel to geo-electric strike is the TE mode. Phase tensor skew angles are less than five degrees for almost all sites at periods up

to 10^2 s, consistent with data being responsive to a 2D regional resistivity structure (Figure S2, S3). Even at longer periods of 10^3 s (Figure S3) most sites are 2D, with a few regions of 3D response.

On the basis that the 110 sites were 2D to at least 10^2 s and most to 10^3 s, we inverted profiles A-A' and B-B' using the WinGlink program[9]. Many inversions were carried out, systematically testing different starting models and smoothing parameters to assess the robustness of the features shown in Figure 2. It was found that smoothing parameter (τ) value of between 1 and 5 resulted in the best balance of data misfit and model smoothness, and an RMS of 1.8 was obtained for $\tau = 1$. The final model has equal smoothing between horizontal and vertical blocks to keep the model simple and avoid introducing geological bias. The robustness of major features was extensively tested by replacing them with resistive features and running inversions and forward modelling, by varying starting half-space resistivity in a systematic way and experimenting with bias in smoothing in either horizontal or vertical directions. Tests showed that the conductive structures C1, C2 and C3 are required to fit the data. The fit between the MT data and the model response resulting from 2D inversions of the preferred models for selected sites is presented in Figure S4. At periods $>10^2$ s, the TM mode is better fitted compared to TE mode as the TM mode is less affected by resistivity variations due to off-transect 3D effects.

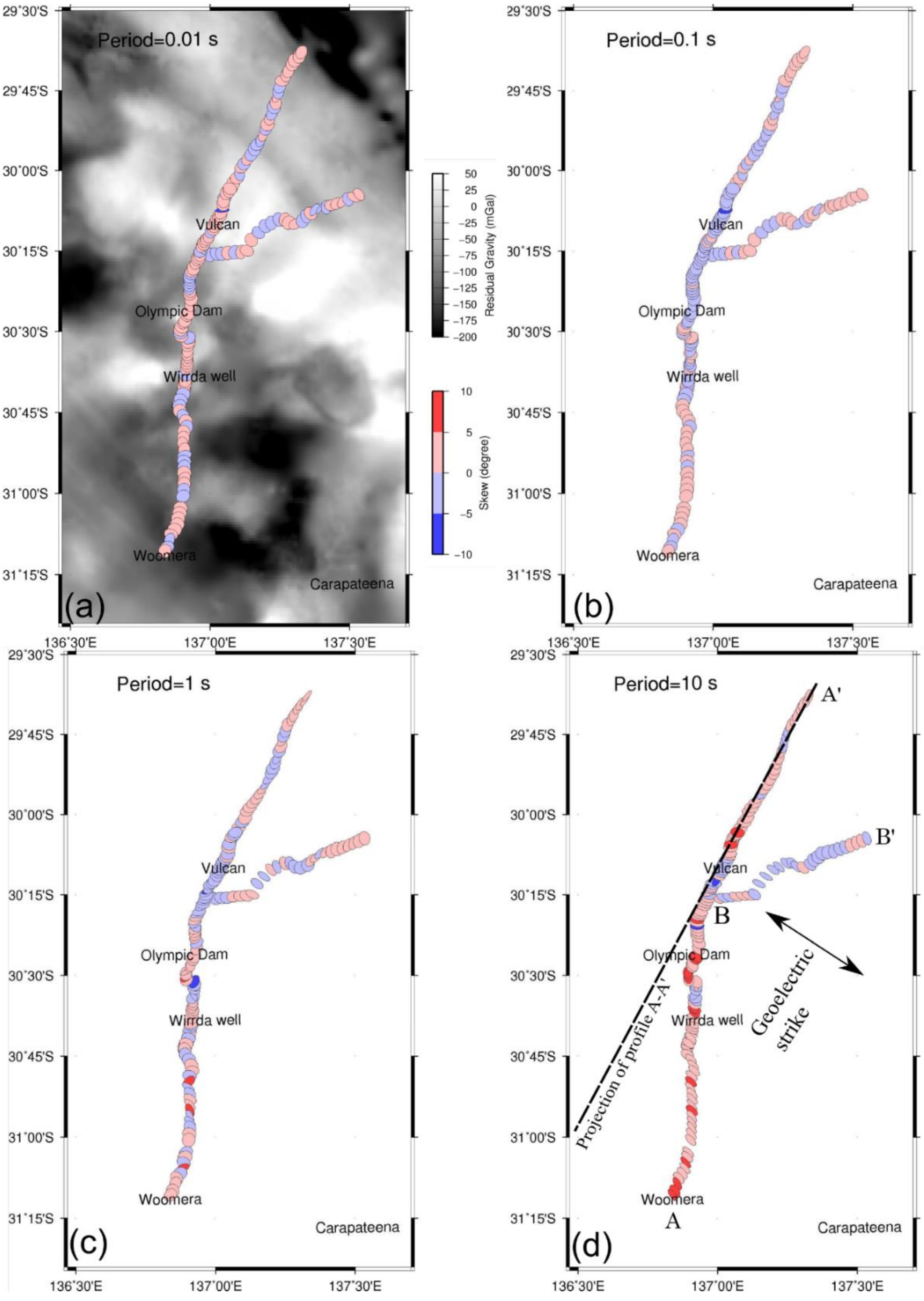

Figure S2: Phase sensor plots at selected periods of (a) 0.01 s, (b) 0.1 s (c) 1 s and (d) 10 s coloured by the skew angle showing that almost all sites have skew angles less than 5 degrees. Skew angles magnitudes greater than 5 degrees are considered 3D. The background image is residual gravity map of the survey area.

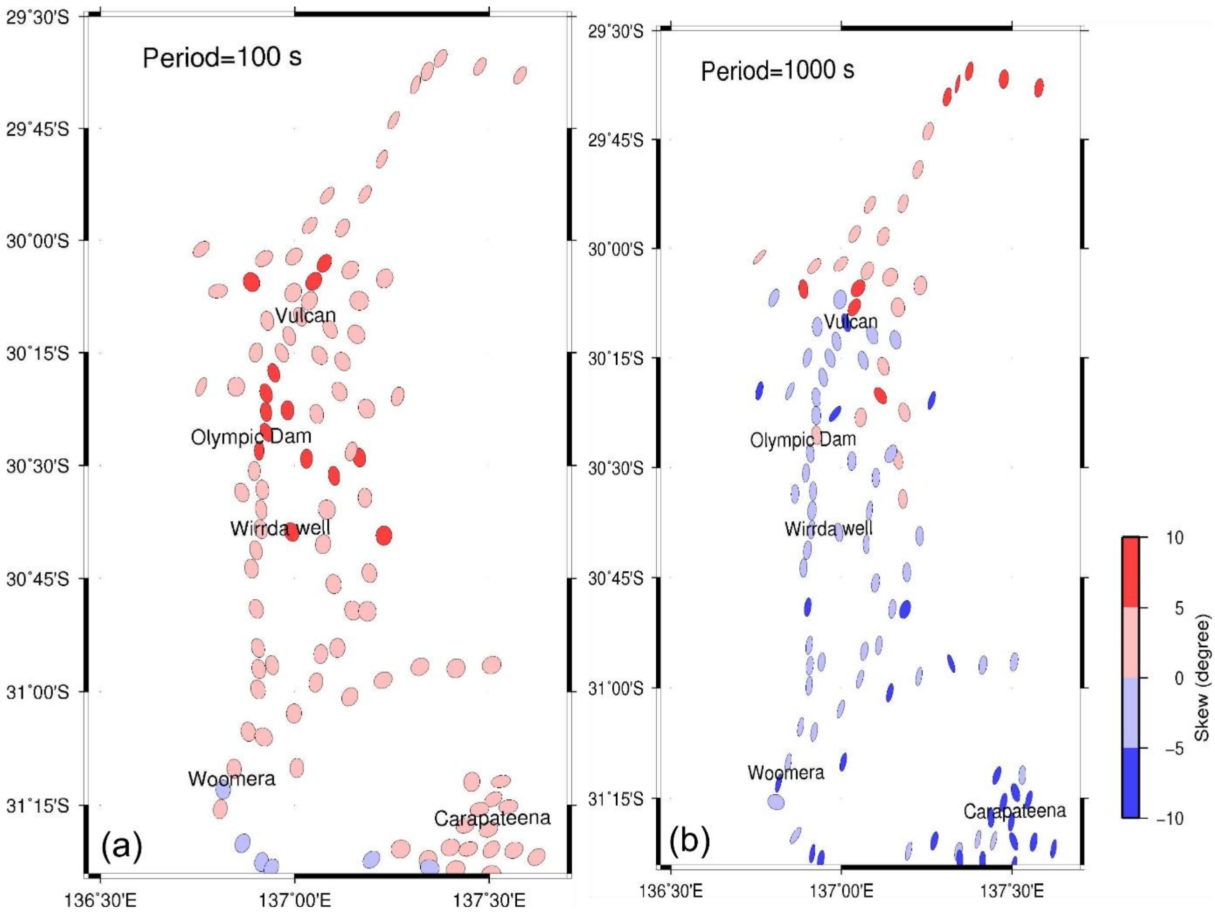

Figure S3: Phase sensor plots at periods of (a) 100 s, (b) 1000 s coloured by the skew angle showing that almost all sites have skew angles less than 5 degrees. Skew angles magnitudes greater than 5 degrees are considered 3D.

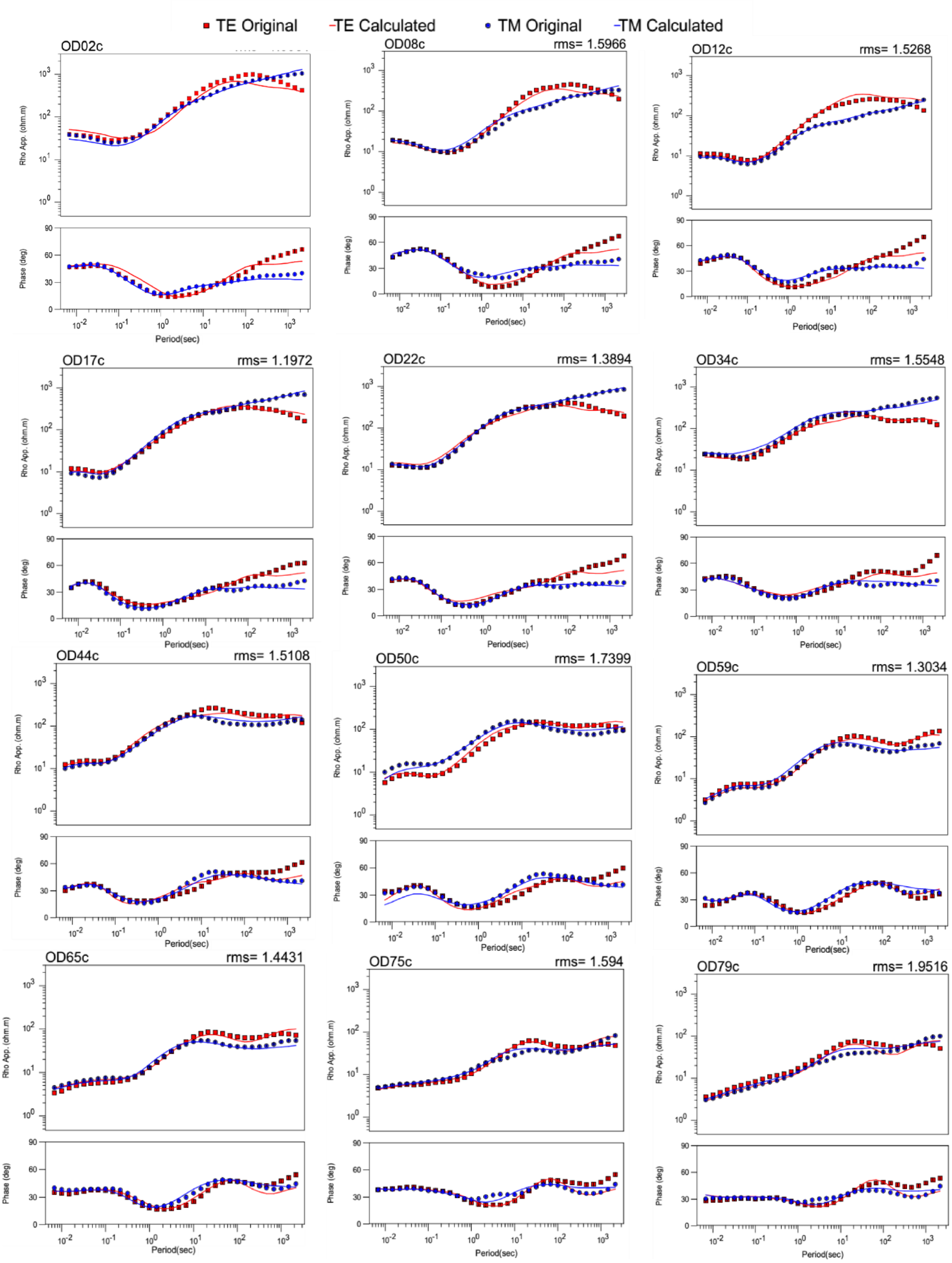

Figure S4: Selected data and model response for 12 sites of the 2D inversion of profile AA' spaced approximately 10 km apart to with total RMS of 1.8. At periods >100 s, the TM mode is better fit as this mode is less affected by resistivity variations due to 3D effects.

The inversion also allowed for static shift on both modes of the apparent resistivity to be determined as an independent variable. Figure S5 shows that static shifts are small, with, less than half-an-order of magnitude spread across all sites. This is expected as the survey area is in a thick sedimentary cover that has relatively uniform low-resistivity.

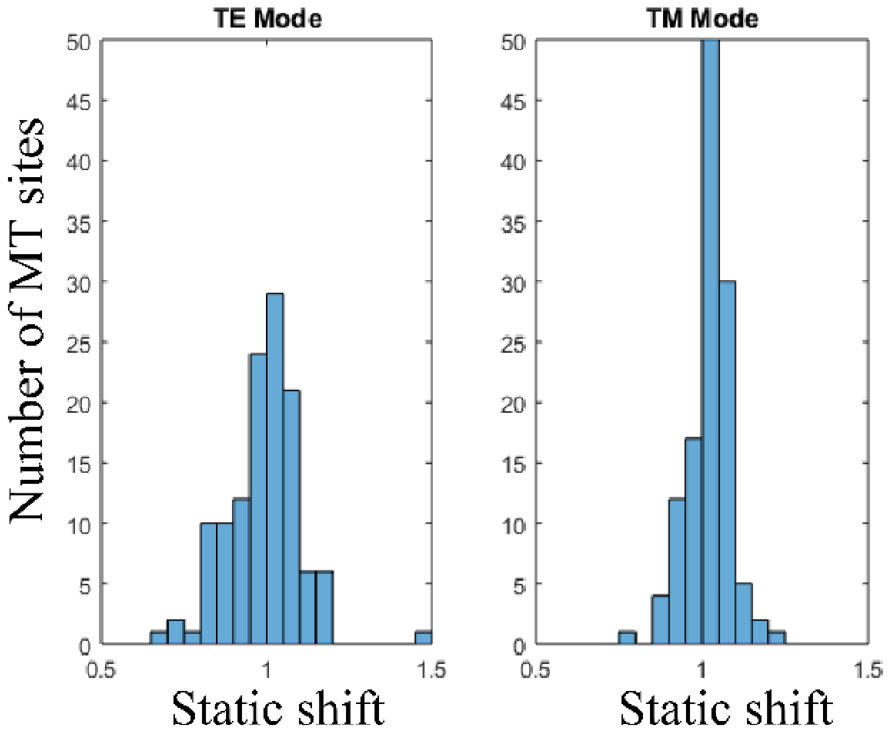

Figure S5: Histogram of static shifts on the apparent resistivity in log units for 110 sites along profile A-A' for the TE mode and TM mode.

An array of long-period MT stations with 5-10 km site spacing, in a 50 km swath around transects A-A' and B-B', encompassed a wider region of exploration interest. We inverted MT data using a 3D inversion code[10] as a check on the plausibility and limitations of the 2D model that necessarily requires infinite strike of structures perpendicular to the transect orientation. The 3D inversion included the full impedance tensor and vertical magnetic transfer function data from 152 stations at 19 periods in the bandwidth of 10^1 - 10^4 s. The model space extends 2400 km × 2200 km × 1000 km in NS, EW and vertical directions, respectively, and the grid

was discretised into 290 × 110 × 58 cells in the x, y and z directions. The central part of the grid has 1250 m horizontal dimensions. Thickness of the first layer is 250 m and increased by a factor of 1.1 for subsequent layers. The starting model was a 100 Ωm half-space. Static shifts were not included in the modelling, but as shown in Figure S5 the shifts are generally small and can be accommodated by thin near-surface mesh blocks between sites. Error floors of 3%, 30% and 0.03 were used for the off-diagonal impedances, diagonal impedances and tipper, respectively. Overall, the RMS fit to all data was 1.7: selected sites of observations and model responses for both apparent resistivity and phase, and for tipper are shown in Figure S6.

A final check on the consistency of the 2D and 3D models was undertaken with an inversion of line B-B'. The model parameterisation was the same as for line A-A' with a smoothing τau=1, and error floors of 5% for apparent resistivity and equivalently 1.43 degrees for phase in both TE and TM modes. Broadband MT responses were again rotated to the dominant geoelectric strike of N115°E but transects was taken along the line of the survey to preserve the geographical extent. Figure S6 shows a low-resistivity zone C3 below 25 km consistent with the 2D and 3D inversions. However, C3 is more resistive that the C3 region under A-A', and it appears that this reflects the limited spatial extent of the conductor in the NW-SE orientation.

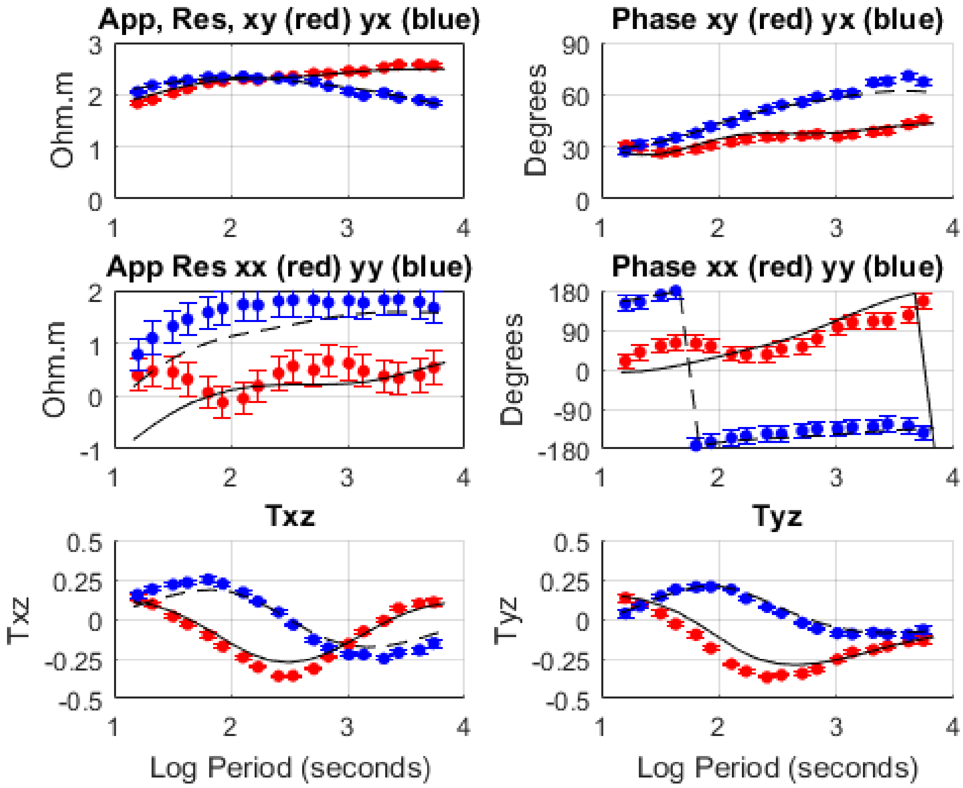
App, Res, xy (red) yx (blue)
Ohm.m
Phase xy (red) yx (blue)
Degrees
App Res xx (red) yy (blue)
Ohm.m
Phase xx (red) yy (blue)
Degrees
Txz
Txz
Tyz
Tyz
Log Period (seconds)
Log Period (seconds)

(a)

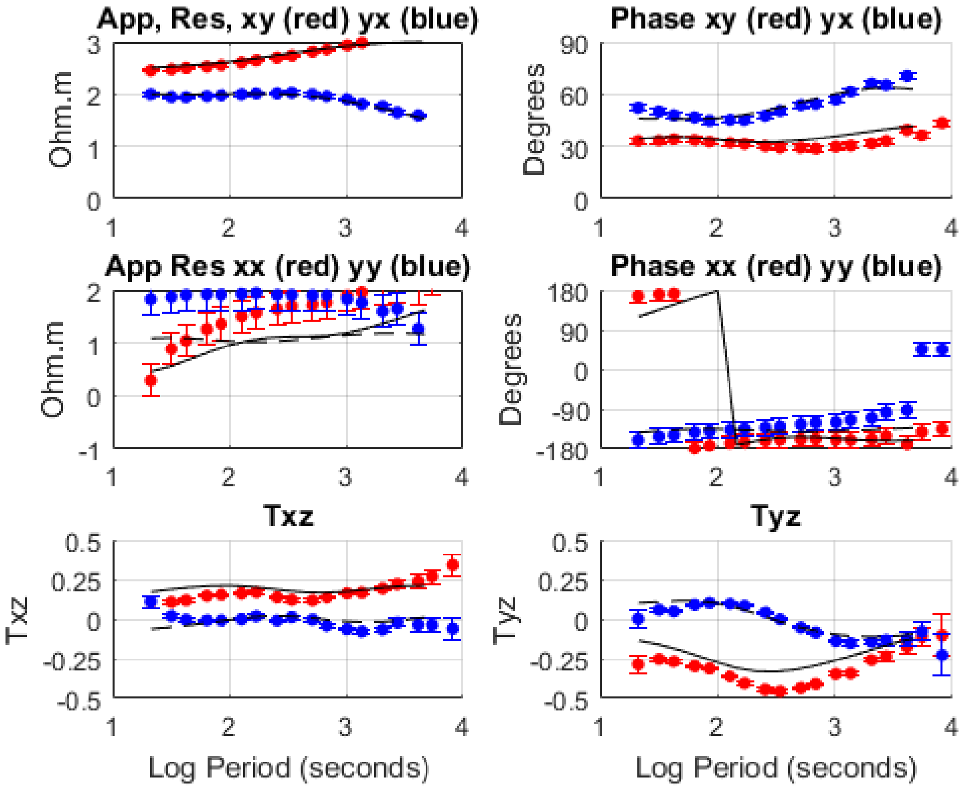
App, Res, xy (red) yx (blue)
Ohm.m
Phase xy (red) yx (blue)
Degrees
App Res xx (red) yy (blue)
Ohm.m
Phase xx (red) yy (blue)
Degrees
Txz
Txz
Tyz
Tyz
Log Period (seconds)
Log Period (seconds)

(b)

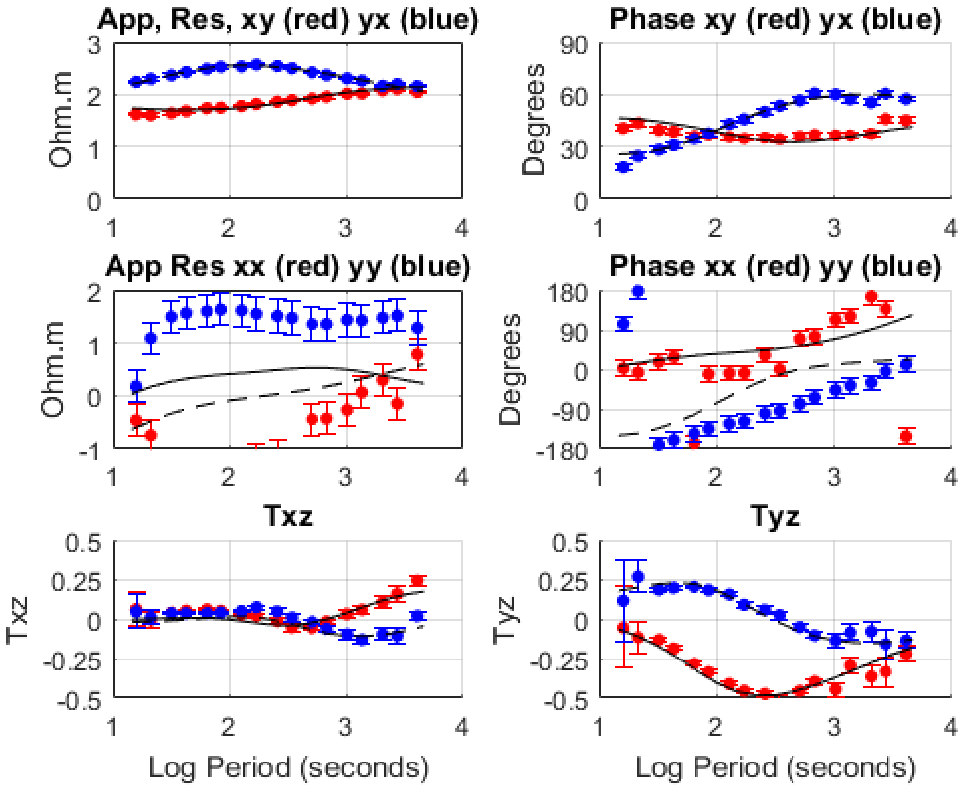

(c)

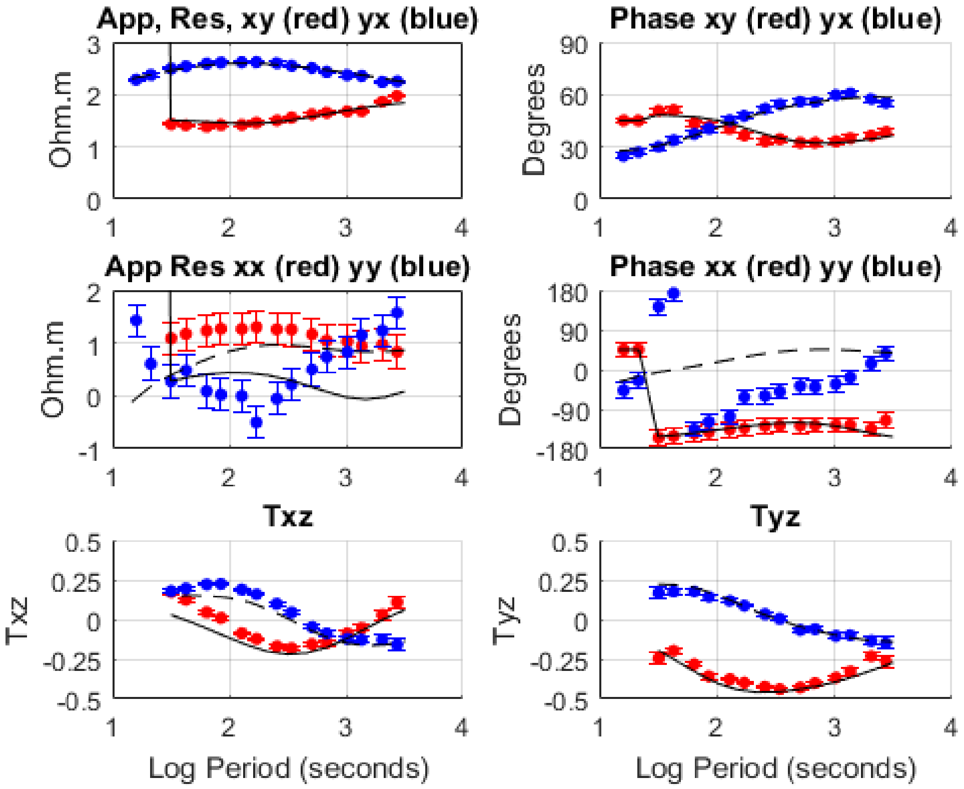

(d)

Figure S6: Selected data and model responses at four sites (a -d); Site (a) is from the western side of the array; site (b) is from the northern side of the array; site (c) is from the southern side

of the array; and site (d) is from the eastern side. For each site the top two panels are for the logarithm of the apparent resistivity (in Ωm) and phase (degrees) for the off-diagonal elements (xy and yx) of the impedance tensor (xy refers to the electric field in geographic north-x direction, and the y to the magnetic field in geographic east-y direction); the middle two panels are for diagonal elements (xx and yy); and the lower two panels are the real (red) and quadrature (blue) parts of the transfer functions between the vertical magnetic field (z) and the horizontal components (x and y). Fits from the 3D inversion are shown as solid and dashed lines.

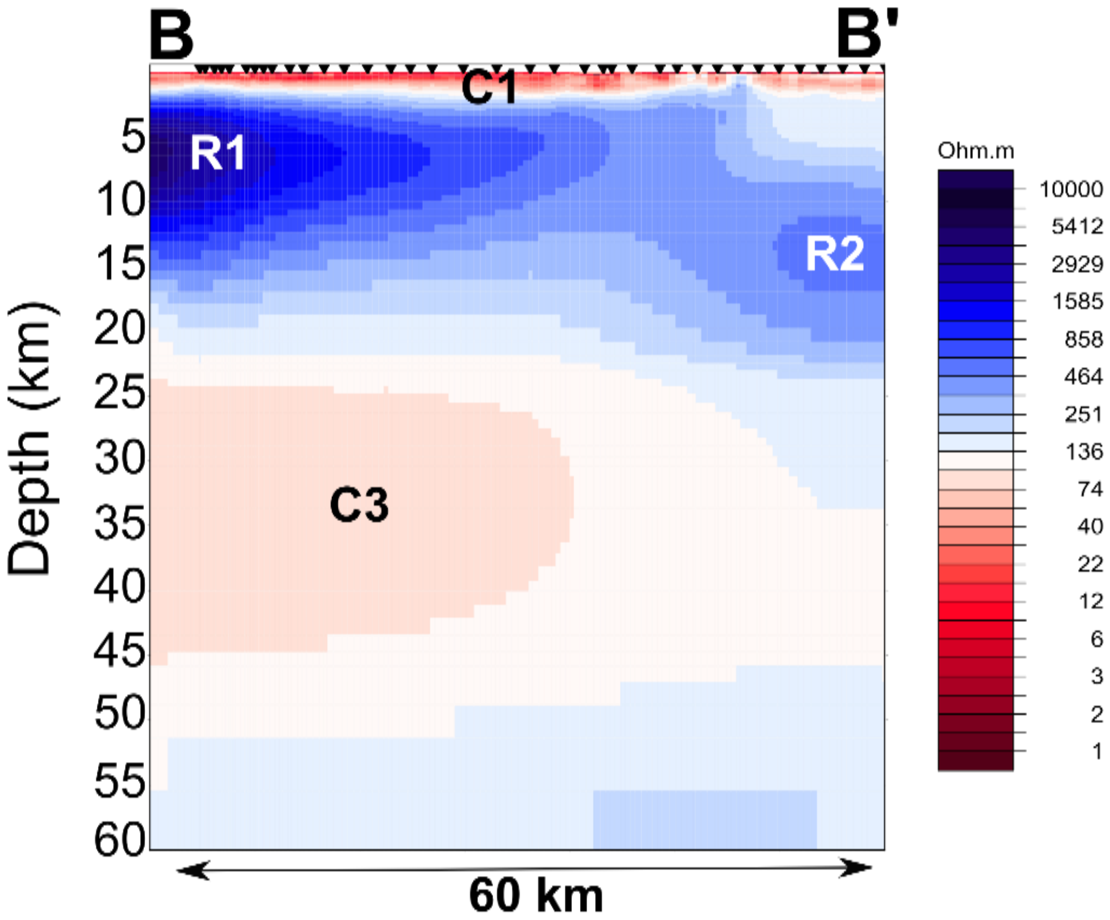

Figure S7: 2D resistivity model of Profile B-B' to a depth of 60 km. The surface conductive layer (C1) is associated with Neo-Proterozoic and Tertiary sediments. The Archean Gawler Craton and Proterozoic mobile belt are characterized by high resistivity (blue colour, R1 and R2). A low-resistivity region (C3) is situated at the margins of the Archean Gawler Craton at a depth 25-45 km in the lower crust and is consistent with the 2D and 3D inversions.

References

1 Wise, T. *et al.* Olympic Dam seismic revisited: reprocessing of deep crustal seismic using partially preserved amplitude processing. *MESA Journal* **78** 17-28 (2015).

2 Drummond, B., Lyons, P., Goleby, B. & Jones, L. Constraining models of the tectonic setting of the giant Olympic Dam iron oxide–copper–gold deposit, South Australia, using deep seismic reflection data. *Tectonophysics* **420**, 91-103, doi:10.1016/j.tecto.2006.01.010 (2006).

3 Chave, A. D. & Jones, A. G. *The magnetotelluric method: Theory and practice*. (Cambridge University Press, 2012).

4 Caldwell, T. G., Bibby, H. M. & Brown, C. The magnetotelluric phase tensor. *Geophysical Journal International* **158**, 457-469, doi:10.1111/j.1365-246X.2004.02281.x (2004).

5 Booker, J. R. The magnetotelluric phase tensor: a critical review. *Surveys in Geophysics* **35**, 7-40, doi:10.1007/s10712-013-9234-2 (2014).

6 Heinson, G. S., Direen, N. G. & Gill, R. M. Magnetotelluric evidence for a deep-crustal mineralizing system beneath the Olympic Dam iron oxide copper-gold deposit, southern Australia. *Geology* **34**, 573-576, doi:10.1130/g22222.1 (2006).

7 Chave, A. D. & Thomson, D. J. Bounded influence magnetotelluric response function estimation. *Geophysical Journal International* **157**, 988-1006, doi:10.1111/j.1365-246X.2004.02203.x (2004).

8 Weaver, J., Agarwal, A. & Lilley, F. Characterization of the magnetotelluric tensor in terms of its invariants. *Geophysical Journal International* **141**, 321-336 (2000).

9 Rodi, W. & Mackie, R. L. Nonlinear conjugate gradients algorithm for 2-D magnetotelluric inversion. *Geophysics* **66**, 174-187 (2001).

10 Mackie, R. L., Rodi, W. & Watts, M. D. in *SEG Technical Program Expanded Abstracts 2001* 1501-1504 (Society of Exploration Geophysicists, 2001).
